# A case report of acute severe paraquat poisoning and long-term follow-up

**DOI:** 10.3892/etm.2014.1727

**Published:** 2014-05-20

**Authors:** GUANGCAI YU, BAOTIAN KAN, XIANGDONG JIAN, JIERU WANG, JING SUN, CHENGZHEN SONG

**Affiliations:** Department of Poisoning and Occupational Diseases, Qilu Hospital of Shandong University, Jinan, Shandong 250012, P.R. China

**Keywords:** acute paraquat poisoning, treatment, long-term follow

## Abstract

In the present study, the successful management of severe paraquat (PQ) poisoning with multiple organ dysfunction syndrome is described. A 42-year-old female ingested >100 ml PQ (20% weight/volume) in an attempted suicide. After 22 h the patient was admitted to hospital with serious liver, kidney and lung damage. Comprehensive therapy that maximized poison elimination was administered, along with appropriate glucocorticoids and medication for anticoagulation and protection of the liver and kidney. The patient was successfully treated and recovered after 40 days. However, pulmonary damage was aggravated when the glucocorticoid treatment was stopped after 2 months; the lungs recovered again following systematic therapy. Subsequent to a 8-month follow-up, the patient was able to look after herself in her daily life. To the best of our knowledge, successful treatment following severe PQ poisoning is rare.

## Introduction

Paraquat (N,N′-dimethyl-4,4′-bipyridinium dichloride; PQ), an effective herbicide that has favorable environmental characteristics, as well as being cost-effective, was first synthesized in 1882 and has been used as an herbicide since 1955 (1). PQ is an important herbicide used in agriculture; however, thousands of individuals succumb due to PQ intoxication every year in the developing world. PQ is a highly toxic compound and the fatality rate of PQ ranges between 60 and 80% (2) due to the lack of a specific antidote. A PQ dose of 30 mg/kg may be fatal, which is equivalent to 8–10 ml of the 20% solution sold commercially (3). PQ has been shown to cause significant damage to organs, including the lung, liver, kidneys and myocardium, with the highest concentration of PQ found in the lungs (4). The prognosis of patients with multiple organ dysfunction syndrome (MODS) caused by fulminant poisoning (>40 mg PQ ion per kg of body weight) is extremely dangerous and patients may succumb within hours to a few days following ingestion (5,6).

China is an agricultural country and PQ is used extensively. Since the first reported case of PQ poisoning, studies have focused on the mechanism and effects of combination therapies and various agents (7,8). Furthermore, strategies for the management of PQ poisoning have focused on the modification of the toxicokinetics of the poison by either decreasing its absorption or enhancing its elimination (9); however, studies on comprehensive strategies are rare. We previously identified a comprehensive treatment strategy against PQ poisoning, termed the Qilu scheme (10). In the present study, a case of MODS caused by severe PQ poisoning was treated using multi-target comprehensive therapy. Furthermore, the present study indicates the potential and feasibility of the therapy for the treatment of PQ poisoning. Informed consent was obtained from the patient. The study was approved by the ethics committee of the Qilu Hospital, Shandong University (Jinan, China).

## Case report

A 42-year-old female, weighing ~60 kg, ingested >100 ml PQ (20% weight/volume) in an attempted suicide at 12:50 p.m. on September 13, 2012. The patient experienced nausea, vomiting and discomfort immediately following PQ ingestion and was sent to a county hospital 20 min later. A gastric lavage was immediately performed and kaolin (30 g) and mannitol infusion (250 ml) was administered within 1 h following ingestion. Hemoperfusion therapy was prescribed for 2 h. The dose for methylprednisolone was 1,000 mg/day for the first day. Due to the severity of the condition, relatives brought the patient to the Department of Poisoning and Occupational Diseases, Qilu Hospital of Shandong University at 10:55 a.m. on December 14, 2012. On admission, the patient’s vital signs were normal, with a body temperature 36.6°C, pulse 89 beats/min, respiratory rate 19 breaths/min, blood pressure 125/80 mmHg and blood oxygen saturation 98%. Physical examination was normal, with the exception of painful erosions in the oral cavity. Based on the typical poison-associated symptoms in the mouth and the gastrointestinal tract, computed tomography (CT) manifestations, significantly abnormal results in the main laboratory test results and the information provided by the patient and her relatives on admission, the patient was diagnosed with severe acute PQ poisoning.

Following admission to Qilu Hospital, the patient was subjected to complete gastrointestinal decontamination using activated carbon, Smecta and mannitol in order to thoroughly remove the pesticide residue from the gastrointestinal tract. Furthermore, other treatments such as an intravenous drip of high doses of glucocorticoids, anticoagulants, anti-free radical, liver- and kidney-protecting and water and electrolyte balance-maintaining agents, and treatment with Chinese herbs (including Xuebijing, *Cordyceps* and depside salt from *Salvia miltiorrhiza*) were administered to the patient. This was termed the Qilu therapeutic schedule. The dose for glucocorticoids was 800 mg/day for the first 4 days and then reduced to 40–80 mg/day. The patient’s condition was markedly improved following the comprehensive therapy. Low doses of prednisone were maintained after 3–6 weeks. After 2 months the patient stopped taking glucocorticoids; however, the pulmonary damage became aggravated and recovered again subsequent to treatment with glucocorticoid and traditional Chinese medicines.

**Figure 1:**
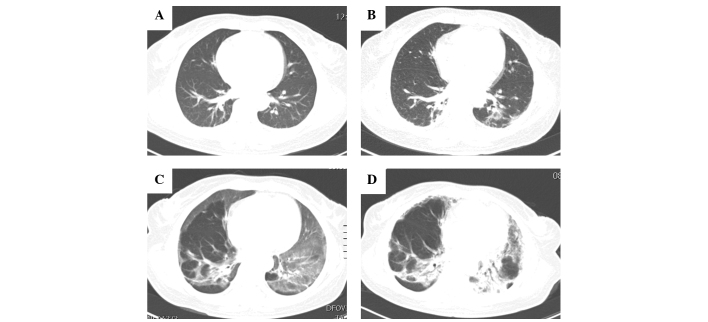
Lung CT scans at different time-points following ingestion of paraquat. (A) Initial CT scan (2 days following ingestion) showing increased lung markings and pleural thickening, with small inflammatory patches observed around the pleura. (B) CT scan 5 days following ingestion. Cotton-wool spots and cloudy high density are visible, primarily in the left lung. (C) A follow up CT scan (15 days following ingestion) demonstrating showing diffuse patchy shadows and ground glass appearance, observed primarily in the left lung. (D) At 24 days following ingestion, the ground-glass attenuation had decreased markedly, fibrotic changes were dominant in the lung. CT, computed tomography.

**Figure 2:**
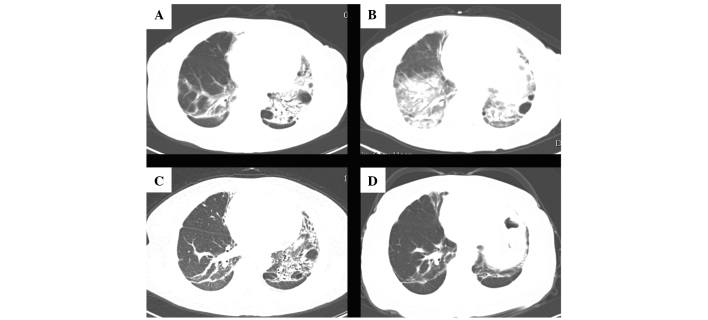
Lung CT scans at different time-points following ingestion of paraquat. (A) At 44 days following ingestion, the lung fibrosis had decreased. (B) At 75 days post-ingestion the lung damage was aggravated. Larger lesions with air and fluid bronchograms, surrounded by a wide range of patchy lesions were observed. (C) A follow up CT scan after 148 days showed that the lung damage had decreased markedly once the treatment was resumed. (D) A CT of the lung 236 days following paraquat exposure revealed localized fibrosis was improved. CT, computed tomography.

Following multi-target comprehensive therapy, the dynamic changes in the lung CT scans are shown in Fig. 1 and 2, and the main laboratory test results are shown in Table I. The liver and kidney functions were seriously damaged 2 days following ingestion and the most serious damage was observed 6 days subsequent to PQ poisoning. However, the liver and kidney functions gradually recovered 2 weeks later (Table I). The initial CT scan showed that there was a certain degree of lung injury that occurred after 2 days (Fig. 1A) and gradually accelerated over the following 3 days (Fig. 1B). Serious lung injury occurred 15 days later (Fig. 1C); however, it improved gradually during the 6 weeks of treatment (Fig. 1D and Fig. 2A). Treatment was ceased gradually; however, after 2 months the lung injury re-occurred (Fig. 2B) accompanied by marked liver dysfunction. Treatment was then resumed and lung function improved again (Fig. 2C), and the liver function gradually returned to normal. Eight months following ingestion, the patient’s lung function was markedly improved (Fig. 2D). The pulmonary function test indicated moderate restrictive ventilation dysfunction and a mild decline of diffuse lung function with the forced vital capacity, maximum vital capacity, maximum ventilatory volume and carbon monoxide diffusion capacity accounting for 53.2, 52.1, 67.3 and 54.1% of the expected values, respectively. The patient continues to be followed up.

## Discussion

PQ is a non-selective herbicide that has been widely used in agriculture since the 1960s (11). Although it has been found to be safe for occupational use, PQ poisoning has been observed in patients who ingest the pesticide either accidentally or intentionally in an attempt to commit suicide. PQ is banned or rarely used in the developed world; however, in developing countries PQ continues to be used and PQ poisoning remains a major cause of mortality among patients with acute poisoning (12). During acute PQ poisoning, the pulmonary concentrations of PQ may be higher than the plasma concentration (13). Therefore, the primary cause of mortality in PQ poisoning is respiratory failure due to oxidative damage to the alveolar epithelium with subsequent obliterating fibrosis (14). In addition to lung damage, PQ ingestion has been shown to injure other organs, but to a lesser extent. Severe PQ poisoning is characterized by multi-organ involvement, primarily affecting the lungs, kidneys, liver, myocardium and adrenal cortex (15). Therefore, urgent gastric lavage is required to reduce the absorption of PQ. Extracorporeal elimination, for example hemoperfusion (HP), is an effective measure that has previously been used clinically (11). The majority of studies have focused on the association between HP and the overall prognosis of PQ poisoning. However, the results from these studies are discouraging and controversial opinions have arisen on the therapeutic effects of HP in PQ intoxication treatment (16,17). The prognosis for patients with severe PQ poisoning, complicated with multiple organ damage, is extremely poor. Despite numerous studies into the mechanism of toxicity and the potential therapeutic treatments for PQ poisoning, at present no specific therapy has been shown to affect the outcome in controlled clinical studies (12).

In the present case study, a patient with PQ poisoning complicated with MODS was successfully treated using multi-target comprehensive therapy. The patient ingested a high dose of PQ, resulting in severe liver and kidney damage. Gastrointestinal procedures were performed to prevent further absorption of PQ residue, accompanied by glucocorticoid treatment to reduce cell absorption, promote the elimination of PQ and control lung inflammation lesions. The mechanism of PQ toxicity has been previously investigated by Dinis-Oliveira *et al* (18). In the present study, anticoagulant and antioxidant treatments, as well as Chinese medicine, were administered to the patient to prevent oxidative damage and to protect other organs against injury. Pulmonary lesions gradually developed into pulmonary fibrosis; therefore, the patient was given the immunosuppressant cyclophosphamide. The patient’s condition was aggravated following the cessation of the treatment. The lung injury was improved once treatment with glucocorticoids was resumed, indicating that glucocorticoids may have an important role in the treatment of pulmonary fibrosis caused by PQ poisoning. The patient was cured and discharged from hospital. However, further studies are required to determine an effective treatment against PQ poisoning.

## Figures and Tables

**Table I:** Changes in the main indicators of blood, urine, liver and kidney function test results for the patient 2, 6, 14, 43, 71, 127 and 221 days following ingestion.

Date	ALT* (U/l)	AST* (U/l)	GGT* (U/l)	Cr (μmol/l)	BUN (mmol/l)	CK-MB* (ng/ml)	CK* (U/l)	DDi* (μg/ml)	WBC (10^9^/l)	HGB (g/l)	PLT (10^9^/l)	ESR (mm/h)	PRO
Day 2	219	212	108	241	13.3	4.2	410	0.79	21.05	140	115	16	1+
Day 6	815	304	797	274	23.9	2.2	117	0.85	12.79	132	162	44	1+
Day 14	53	39	316	53	5.4	5.2	75	0.70	20.76	115	369	69	1+
Day 43	171	82	610	41	5.7	2.5	29	0.16	9.08	118	199	40	−
Day 71	27	24	156	40	1.8	1.5	28	0.64	5.95	135	220	27	+−
Day 127	29	20	58	57	4.4	2.0	31	0.54	11.39	128	236	13	−
Day 221	23	22	49	60	2.6	0.8	64	0.30	5.74	125	207	2	−

* Reference values: ALT, AST (0–40 U/l); CK-MB (0.3–4.0 ng/ml); CK (26–140 U/l); DDi (0–0.5 μg/ml); GGT (3–50 U/l). Blood biochemistry was tested using a Roche cobas^®^ 8000 automatic biochemical analyzer; blood routine, urine routine and blood sedimentation was tested using the Sysmex XE-2100 automatic biochemical analyzer; DDi was tested using Beckman Kurt’s ACL TOP 700 automatic blood coagulation analyzer.

ALT, alanine aminotransferase; AST, aspartate aminotransferase; GGT, glutathione-*S*-transferase; CR, creatinine; BUN, blood urea nitrogen; CK-MB, creatine kinase MB isoenzyme; DDi, D-dimer; WBC, white blood cell; HGB, hemoglobin; PLT, platelet; ESR, erythrocyte sedimentation rate; PRO, urine protein. The values 1+, − and +-indicate positive and suspicious positive.
